# Heart failure subtype after acute kidney injury

**DOI:** 10.1186/s12882-024-03602-1

**Published:** 2024-05-17

**Authors:** Bethany C. Birkelo, Evan Brittain, Andrew Guide, Robert A. Greevy, Michael E. Matheny, Jeffrey Annis, Trey Richardson, Sarah Faubel, Edward D. Siew

**Affiliations:** 1 Division of Nephrology and Hypertension, University of Minnesota, 717 Delaware St. SE, Minneapolis, MN 55414 USA; 2 Division of Cardiovascular Medicine, Vanderbilt University Medical Center, Nashville, TN USA; 3 Department of Biostatistics, Vanderbilt University Medical Center, Nashville, TN USA; 4 Division of Nephrology and Hypertension, Vanderbilt Center for Kidney Disease (VCKD) and Integrated Program for Acute Kidney Injury Research (VIP-AKI), Vanderbilt University Medical Center, Nashville, TN USA; 5 VA Tennessee Valley, Health Services Research and Development, Nashville, USA; 6 Department of Biomedical Informatics, Vanderbilt University Medical Center, Nashville, TN USA; 7 VA Geriatrics Research Education and Clinical Center (GRECC), Tennessee Valley Health System (THVS), Veteran’s Health Administration, Nashville, TN USA; 8 Vanderbilt Institute for Clinical and Translational Research, Vanderbilt University Medical Center, Nashville, TN USA; 9 Department of Medicine, University of Colorado School of Medicine, Aurora, CO USA

**Keywords:** Acute kidney injury, Cardiovascular outcomes, Heart failure

## Abstract

**Introduction:**

Acute kidney injury (AKI) is associated with increased risk of heart failure (HF). Determining the type of HF experienced by AKI survivors (heart failure with preserved or reduced ejection fraction, HFpEF or HFrEF) could suggest potential mechanisms underlying the association and opportunities for improving post-AKI care.

**Methods:**

In this retrospective study of adults within the Vanderbilt University health system with a diagnosis of HF, we tested whether AKI events in the two years preceding incident HF associated more with HFpEF or HFrEF while controlling for known predictors. HF outcomes were defined by administrative codes and classified as HFpEF or HFrEF by echocardiogram data. We used multivariable logistic regression models to estimate the effects of AKI on the odds of incident HFpEF versus HFrEF.

**Results:**

AKI (all stages) trended towards a preferential association with HFpEF in adjusted analyses (adjusted OR 0.80, 95% CI 0.63 – 1.01). Stage 1 AKI was associated with higher odds of HFpEF that was statistically significant (adjusted OR 0.62, 95% CI 0.43 – 0.88), whereas stages 2–3 AKI showed a trend toward HFrEF that did not reach statistical significance (adjusted OR 1.11, 95% CI 0.76 – 1.63).

**Conclusions:**

AKI as a binary outcome trended towards a preferential association with HFpEF. Stage 1 AKI was associated with higher odds of HFpEF, whereas stage 2–3 trended towards an association with HFrEF that did not meet statistical significance. Different mechanisms may predominate in incident HF following mild versus more severe AKI. Close follow-up with particular attention to volume status and cardiac function after discharge is warranted after even mild AKI.

## Introduction

Heart failure (HF) comprises an enormous burden on human health, afflicting over 64 million people globally [1]. Despite considerable overlap in risk factors, distinct pathophysiologic processes underlie HF with preserved ejection fraction (HFpEF) versus HF with reduced ejection fraction (HFrEF). While HFrEF results from a progressive loss of cardiomyocytes, often following cardiac ischemia, HFpEF is thought to be caused by multimorbidity-induced systemic inflammation that results in cardiomyocyte stiffness and fibrosis via coronary microvascular endothelial inflammation [2]. The high prevalence of comorbidities in patients with HFpEF support this paradigm [3].

Patients with HF commonly experience kidney dysfunction before and/or after they are diagnosed with HF. HF and kidney disease exist in a complex, bidirectional relationship in which dysfunction in one organ can cause or result from dysfunction in the other. Of the types of kidney disease leading to HF, chronic kidney disease (CKD) is the most well-described; up to one-fifth of patients with CKD go on to develop incident HF [4]. The association between CKD and HFpEF is particularly strong, with an independent association between the two conditions shown in observational data [5] and abnormalities in cardiac mechanics common to both conditions [6, 7]. Acute kidney injury (AKI) is increasingly recognized as a risk factor for HF. Cardiac changes after AKI have been observed in preclinical studies [8, 9], and observational studies have shown increased risk of HF hospitalizations [10] and incident HF [11] after AKI. The predominant type of HF that occurs after AKI (HFpEF versus HFrEF) is not known. Understanding HF type after AKI could suggest possible mechanisms of this aspect of cardiorenal syndrome and highlight areas of focus for post-AKI care. We hypothesized that AKI would be more strongly associated with incident HFpEF compared to HFrEF. To test this hypothesis, we used a population of HF patients to test whether AKI in the two years prior selectively associates with HFpEF or HFrEF.

## Methods

### Study setting and design

In this single center retrospective study, we compared the odds of incident HFpEF versus HFrEF after AKI among adults who obtained regular medical care (at least 3 visits within 5 years) through the Vanderbilt University health system between 2008 and 2022. We ascertained AKI events occurring in the two years prior to HF and adjusted for baseline comorbidities, vitals, and medications prior to the AKI time window (i.e., greater than two years prior to HF). Our choice of a two-year ascertainment period for AKI was informed by preclinical data demonstrating cardiac changes in the heart occurring within days of AKI, and we wanted to focus on the acute effect as opposed to a longer time period, which we would expect to result in a higher proportion of new onset CKD from unrecovered AKI events. The missing echocardiogram data presents a challenge in testing for a preferential association of AKI with HFpEF or HFrEF, so we focused on patients with available echocardiograms by utilizing a study design akin to a case–control design. A strength of the case–control design is the prevalence of the outcome in the sample does not need to reflect the prevalence in the general population in order to accurately estimate the odds ratios of interest. In this study design, patients with HFrEF are “cases” and those with HFpEF are “controls”, which allows the estimation of odds ratios to indicate whether each variable in a logistic model associates more strongly with HFrEF or HFpEF. For example, if AKI did not preferentially associate more with HFrEF or HFpEF, the odds ratio for AKI from the logistic model would be close to one. Conversely, an odds ratio significantly greater than one would indicate a preferential association with AKI and HFrEF; and significantly less than one would indicate a preferential association with AKI and HFpEF.

Data were obtained from the Synthetic Derivative (SD), a de-identified mirror image of the electronic health record (EHR) at Vanderbilt University Medical Center which contains date-shifted data from approximately 2.2 million unique patients, including demographics, diagnosis and procedure codes, medications, lab values, procedure reports, and clinical notes. Data in the SD is extracted from the prior homegrown EHR and from Epic since its go-live in 2017 at Vanderbilt University Medical Center (VUMC). This study was approved by the Institutional Review Board at VUMC. Informed consent was waived by the VUMC IRB due to the use of de-identified data which is considered non-human subjects research.

### Eligibility criteria

**Fig. 1:**
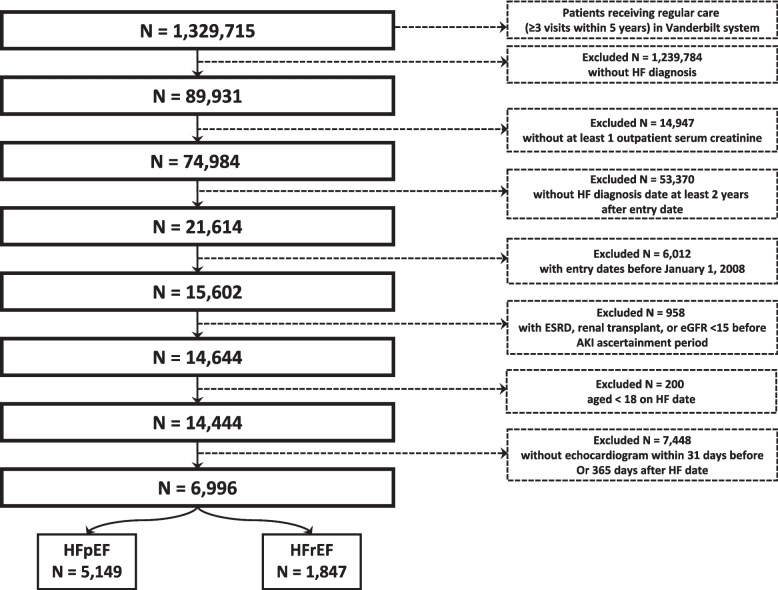
Consort diagram. Eligibility criteria applied to derive final study population

See Fig. 1. The source population consisted of 1,329,715 patients who obtained regular care (≥ 3 outpatient visits in 5 years) within the Vanderbilt University health system. Of those, we identified 89,391 with a HF diagnosis. Inclusion criteria included at least one outpatient serum creatinine value occurring between 2008 and 2020 and at least three years prior to HF diagnosis. Exclusion criteria included baseline eGFR < 15 ml/min/1.37m^2^, kidney transplantation, end-stage renal disease (ESRD), or receipt of dialysis prior to the AKI ascertainment window, and age < 18 years at HF diagnosis. To ensure all HF could be classified as HFpEF or HFrEF, patients without an echocardiogram within 31 days prior to or 365 days after HF diagnosis date were also excluded.

### Data collection

**Fig. 2:**
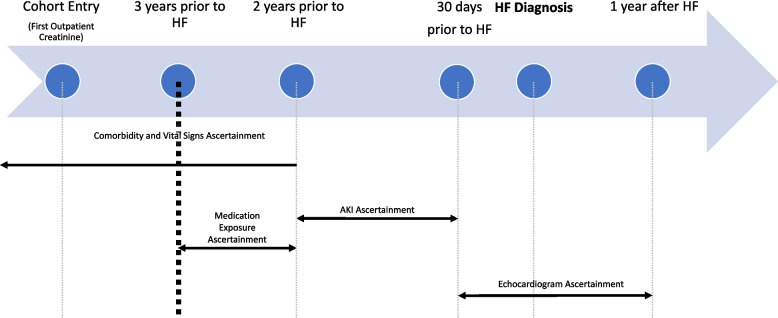
Ascertainment timeline. Time periods of ascertainment of baseline conditions, vital signs, medications, and exposure (AKI events) in relation to outcome (HF diagnosis date). Echocardiogram used to classify HF as HFpEF or HFrEF ascertained in 30 days before and up to 1 year following HF diagnosis date

Baseline comorbidity and vitals data were obtained from records occurring greater than two years prior to the outcome date (date of HF diagnosis, see Fig. 2). Identification of comorbidities and prevalent HF extended back to all available data. To capture active medication use, baseline medications were obtained from prescription information occurs between two and three years prior to outcome date. Exposure data (inpatient serum creatinine values) were obtained from hospitalization records 31 – 730 days prior to the outcome date. Exposure data was not captured in the 30 days prior to outcome date to exclude AKI that occurred concurrently with acute HF. HF outcomes were obtained from diagnosis codes and echocardiogram data (see Definitions for additional details). Diagnosis and procedure codes, which were used to define the HF outcome and baseline conditions, were defined using International Classification of Diseases (ICD) versions 9 (ICD-9) and 10 (ICD-10) and Current Procedural Terminology (CPT) Codes.

### Definitions

#### Cases and controls

We defined cases as HFrEF and controls as HFpEF. A HF diagnosis was defined by either one inpatient diagnosis code or two outpatient diagnosis codes for HF or cardiomyopathy. The date of HF diagnosis was defined as the date of the first inpatient HF diagnosis code or the first of two outpatient diagnosis codes. HF was classified as HFpEF or HFrEF based on the ejection fraction on the echocardiogram closest in time to the HF diagnosis date (HFpEF > 45%, HFrEF ≤ 45%).

### Exposure

The exposure was hospitalized AKI, which was defined using inpatient serum creatinine values and staged using modified Kidney Disease Improving Global Outcomes creatinine-based criteria: stage 1, ≥ 0.3 mg/dl creatinine increase from baseline or creatinine 1.5 to 1.9 times baseline; stage 2, creatinine 2.0 to 2.9 times baseline; and stage 3, creatinine 3.0 times baseline or initiation of dialysis [12]. Baseline serum creatinine was defined as the mean outpatient serum creatinine value occurring 7–365 days prior to the AKI hospitalization. For those without an available baseline in the prior year, the lowest serum creatinine during the AKI hospitalization was used. Estimated glomerular filtration rate (eGFR) was calculated using the Chronic Kidney Disease Epidemiology Collaboration (CKD-EPI) Equation [13]. For patients with more than one AKI event during the ascertainment period, the most severe AKI was used in the analysis.

### Covariates

Baseline comorbidities, vital signs, and laboratory results were ascertained up to two years prior to outcome date. Preadmission medication use was ascertained from two to three years prior to outcome date.

### Statistical approach

Descriptive statistics were obtained by reporting means and standard deviations for continuous covariates, and counts and percentages for categorical data; additionally, the descriptive data was split by HFpEF versus HFrEF. Multivariable logistic regression models were used to estimate the effects of AKI on incident (new onset) HFpEF versus HFrEF, with an odds ratio < 1 indicating greater odds of HFpEF and > 1 indicating greater odds of HFrEF. The primary exposure of interest was AKI. In the primary analysis, AKI was considered a binary variable. In secondary analyses, AKI was modelled as stage 1 and stages 2 or 3 (combined) to consider separately AKI events more likely to be prerenal azotemia (stage 1) versus those more likely to represent parenchymal damage (stages 2–3). To control for confounding, the models included 37 covariates which were ascertained prior to the AKI ascertainment window (see Fig. 2) and included demographics, baseline conditions, and medications. Continuous variables were modelled using three-knot restricted cubic splines to account for their non-linear effects in the models. To address missingness, multiple imputation utilizing predictive mean matching and 5–100 imputations-iterations was used. Confidence intervals were obtained using Wald tests. *P* values were obtained using chi-square tests. All analyses were conducted using R version 4.3.0.

We conducted several sensitivity analyses. First, to ensure that our results were not materially affected by misclassification of the outcome, we used a highly specific definition of HF that required an elevated BNP level (> 100 pg/ml) and a diuretic prescription occurring within 30 days of the HF diagnosis code date. Second, we conducted an analysis limited to patients with HF defined by an inpatient diagnosis code, which we expect to have a higher positive predictive value for HF compared to outpatient codes. Third, because of the high proportion of patients with a history of cancer and the lack of data on chemotherapy drug exposure, we conducted an analysis limited to patients without a history of cancer to ensure our results were not impacted by differential rates of exposure to cardiotoxic agents. Fourth, given the substantial population of adult congenital heart disease patients within the Vanderbilt Health system, we conducted an analysis limited to patients ages 30 years or older. Fifth, due to the degree of missingness of smoking status (missing in 67.5% of patients), we conducted an analysis using imputed smoking status. Sixth, we conducted an analysis limited to patients with EF < 40% or > 50% (thus excluding patients with heart failure with moderately reduced EF). Finally, to evaluate for differences in outcomes in men versus women, we replicated the primary analysis stratified by sex. The opposite directionality of the point estimates for stage 1 AKI versus stages 2–3 AKI in the secondary analyses motivated a post hoc analysis to evaluate for an effect of severe AKI in excess to the overall AKI effect using a model that included stages 1–3 AKI and stages 2–3 AKI as covariates. This model estimates an overall AKI association and a separate additional stage 2–3 AKI association.

## Results

### Patient characteristics

We identified 6,996 patients with incident HF (5,149 with HFpEF and 1,847 with HFrEF) (See Table 1). At baseline, patients who developed HFpEF tended to be older, more often female, and more commonly had atrial fibrillation, anemia, COPD, hypertension, liver disease, and cancer, while those who developed HFrEF had higher prevalence of coronary artery disease and prior myocardial infarction (MI). Baseline eGFR was similar between both groups.

**Table 1:** Patient characteristics at baseline (ascertained up to 2 years prior to HF)

Variable	HFpEF	HFrEF	Overall	*P*-value
Count (N)	5149	1847	6996	
Age at heart failure: Mean (SD)	65.71 (14.54)	64.71 (14.60)	65.44 (14.57)	0.01
Female Sex at Birth: % (N)	52.2% (2687)	39.4% (728)	48.8% (3415)	< 0.01
Race: % (N)				0.03
Black	14.6% (754)	17.1% (316)	15.3% (1070)	
Other^a^	2.7% (137)	2.3% (43)	2.6% (180)	
White	82.7% (4258)	80.6% (1488)	82.1% (5746)	
Albumin: Mean (SD)	4.00 (0.45)	4.00 (0.47)	4.00 (0.46)	0.87
Systolic Blood Pressure: Mean (SD)	133.06 (20.53)	133.28 (21.17)	133.12 (20.70)	0.71
Diastolic Blood Pressure: Mean (SD)	73.95 (12.91)	76.09 (13.71)	74.52 (13.16)	< 0.01
Body Mass Index: Mean (SD)	31.22 (7.99)	29.59 (7.23)	30.80 (7.83)	< 0.01
Estimated GFR: Mean (SD)	82.43 (28.76)	83.45 (28.66)	82.70 (28.73)	0.19
Serum Creatinine: Mean (SD)	0.99 (0.36)	1.02 (0.39)	1.00 (0.37)	< 0.01
Atrial Fibrillation: % (N)	17.8% (915)	13.5% (250)	16.7% (1165)	< 0.01
Anemia: % (N)	9.6% (496)	7.1% (132)	9.0% (628)	< 0.01
COPD: % (N)	18.7% (962)	15.4% (284)	17.8% (1246)	< 0.01
Coronary Artery Disease: % (N)	31.4% (1617)	34.1% (629)	32.1% (2246)	0.04
Diabetes: % (N)	30.9% (1589)	30.3% (560)	30.7% (2149)	0.69
Hypertension: % (N)	65.9% (3394)	60.4% (1115)	64.5% (4509)	< 0.01
Liver Disease: % (N)	8.6% (442)	4.4% (81)	7.5% (523)	< 0.01
Myocardial Infarction: % (N)	8.4% (430)	9.9% (182)	8.7% (612)	0.06
Peripheral Artery Disease: % (N)	9.1% (468)	8.1% (149)	8.8% (617)	0.20
Rheumatic Disease: % (N)	7.2% (371)	6.0% (111)	6.9% (482)	0.09
Alcohol Abuse: % (N)	3.0% (156)	3.5% (64)	3.1% (220)	0.40
Smoking Status: % (N)				0.01
Former	8.0% (414)	6.2% (114)	7.5% (528)	
Never	21.2% (1093)	16.1% (298)	19.9% (1391)	
Smoker	4.1% (213)	5.0% (92)	4.4% (305)	
Unknown	0.8% (41)	0.6% (12)	0.8% (53)	
Missing	65.8% (3388)	72.1% (1331)	67.5% (4719)	
Cancer: % (N)	25.1% (1292)	23.3% (430)	24.6% (1722)	0.13
HIV: % (N)	1.8% (92)	2.2% (40)	1.9% (132)	0.35
Dyslipidemia: % (N)	70.7% (3640)	69.7% (1288)	70.4% (4928)	0.46
CT Scan with Contrast: % (N)	2.5% (127)	1.7% (31)	2.3% (158)	0.06
Ace Inhibitors: % (N)	20.6% (1063)	19.1% (353)	20.2% (1416)	0.17
Alpha Blockers: % (N)	2.0% (102)	1.2% (23)	1.8% (125)	0.05
Beta Blockers: % (N)	25.6% (1319)	19.7% (364)	24.1% (1683)	< 0.01
Loop Diuretics: % (N)	14.8% (760)	7.6% (140)	12.9% (900)	< 0.01
Mineralocorticoid Antagonists: % (N)	3.2% (165)	1.7% (31)	2.8% (196)	< 0.01
Nitrates: % (N)	8.8% (453)	7.1% (132)	8.4% (585)	0.03
Non-Loop Diuretics: % (N)	18.1% (931)	14.3% (265)	17.1% (1196)	< 0.01
Statins: % (N)	28.6% (1471)	24.7% (456)	27.5% (1927)	< 0.01
NSAIDS: % (N)	20.4% (1051)	16.9% (313)	19.5% (1364)	< 0.01
Calcium Channel Blockers: % (N)	20.6% (1060)	15.6% (289)	19.3% (1349)	< 0.01
Angiotensin II Receptor Antagonists: % (N)	14.3% (736)	9.9% (183)	13.1% (919)	< 0.01
Insulin: % (N)	8.5% (436)	6.1% (112)	7.8% (548)	< 0.01
Metformin: % (N)	10.1% (520)	8.9% (164)	9.8% (684)	0.14
Other Diabetes Medications: % (N)	8.2% (424)	7.9% (146)	8.1% (570)	0.69

Covariates had no missingness except for albumin (19% missing in HFpEF, 24% missing in HFrEF), systolic BP (< 1% missing in HFpEF), diastolic BP (< 1% missing in HFpEF), BMI (15% missing in HFpEF, 18% missing in HFrEF), eGFR (< 1% missing in HFpEF and HFrEF), and smoking status (65.8% missing in HFpEF, 72.1% missing in HFrEF)

^a^Other Race includes Asian, Alaskan/Indian, Native Hawaiian/Pacific Islander, unknown, or declined to answer

### Exposure

An AKI event occurred during the ascertainment period in 6.9% of those who developed HFpEF and 5.7% of those who developed HFrEF (see Table 2). The overall distribution of AKI severity was higher among those who developed HFrEF (61%, 20%, and 19% for stages 1, 2, and 3, respectively) versus HFpEF (72%, 16%, and 13% for stages 1, 2, and 3, respectively, *P* = 0.062) though the difference was not statistically significant.

**Table 2:** Rates of AKI in the two years prior to HF

	**HFpEF**	**HFrEF**	**Overall**	***P*-value**
AKI: % (N)	6.9% (355)	5.7% (105)	6.6% (460)	0.08
AKI Stage: % (N)				
0	93.1% (4794)	94.3% (1742)	93.4% (6536)	0.06
1	4.9% (254)	3.5% (64)	4.5% (318)	
2	1.1% (56)	1.1% (21)	1.1% (77)	
3	0.9% (45)	1.1% (20)	0.9% (65)	

### Outcomes

**Fig. 3:**
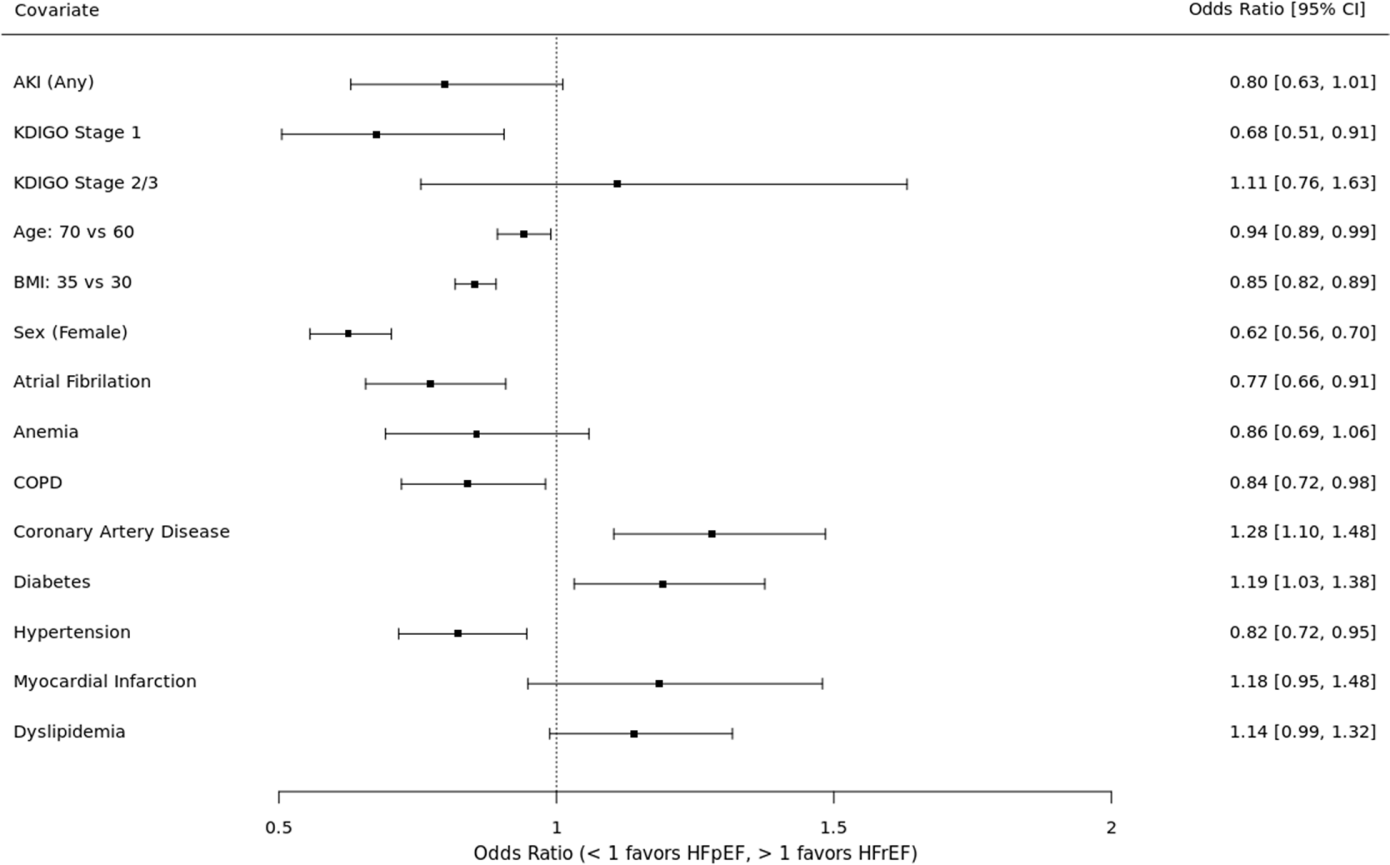
Risk of HFpEF versus HFrEF. Results of primary analysis. Odds ratios for each covariate indicate higher odds of HFpEF (OR < 1) or HFrEF (OR > 1)

See Fig. 3. In the unadjusted analysis, AKI was not preferentially associated with HF subtype (OR 0.81, 95% CI 0.65 – 1.02). When examined by AKI severity, stage 1 AKI was associated with 31% higher odds of HFpEF (OR 0.69, 95% CI 0.52 – 0.92) whereas stage 2–3 had a non-significant trend towards HFrEF (OR 1.12, 95% CI 0.77 – 1.61).

In the adjusted analysis, AKI trended toward an association with HFpEF (OR 0.80, 95% CI 0.63 – 1.01) but did not meet statistical significance. When examined by AKI severity, stage 1 AKI was associated with 32% higher odds of HFpEF (OR 0.68, 95% CI 0.51 – 0.91) whereas stage 2–3 had a non-significant trend towards HFrEF (OR 1.11, 95% CI 0.76 – 1.63). The covariates with statistically significant associations with HFpEF included older age, higher BMI, female sex, atrial fibrillation, and hypertension. The covariates with significant associations with HFrEF included coronary artery disease and black race.

### Sensitivity analyses

#### HF defined by diagnosis code + BNP + diuretic

**Fig. 4:**
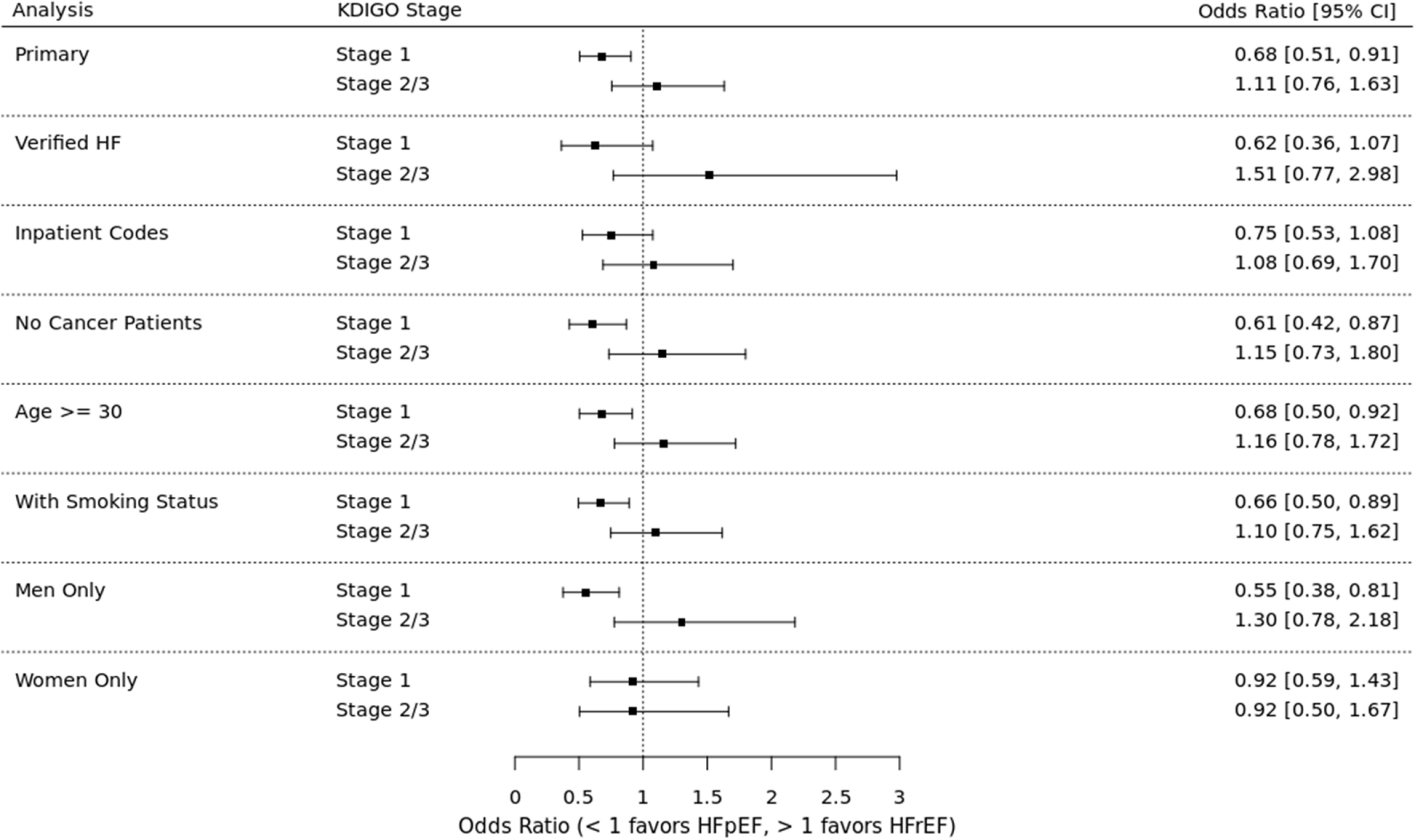
Risks of HFpEF versus HFrEF in primary analysis and sensitivity analyses. Odds ratios for each analysis indicate higher odds of HFpEF (OR < 1) or HFrEF (OR > 1)

See Fig. 4 for sensitivity analyses results. Among patients for whom BNP and diuretic data were available, AKI (all stages) was associated with 13% higher odds of HFpEF (OR 0.87, 95% CI 0.56 – 1.33). Stage 1 AKI was associated with 38% higher odds of HFpEF (OR 0.62, 95% CI 0.36 – 1.07) and stage 2–3 AKI was associated with 51% higher odds of HFrEF (OR 1.51, 95% CI 0.77 – 2.98).

#### HF defined by only inpatient diagnosis code

Among patients in whom HF was diagnosed by an inpatient diagnosis code, AKI (all stages) was associated with 14% higher odds of HFpEF (OR 0.86, 95% CI 0.64 – 1.14). Stage 1 AKI was associated with 25% higher odds of HFpEF (OR 0.75, 95% CI 0.53 – 1.08) and stage 2–3 AKI was associated with 8% higher odds of HFrEF (OR 1.08, 95% CI 0.69 – 1.70).

#### Patients without cancer

Among patients without a history of cancer, AKI (all stages) was associated with 24% higher odds of HFpEF (OR 0.76, 95% CI 0.57 – 1.01). Stage 1 AKI was associated with 39% higher odds of HFpEF (OR 0.61, 95% CI 0.42 – 0.87) and stage 2–3 AKI was associated with 15% higher odds of HFrEF (OR 1.15, 95% CI 0.73 – 1.80).

#### Patients > 30 years of age

Among patients aged 30 years or older, AKI (all stages) was associated with 19% higher odds of HFpEF (OR 0.81, 95% CI 0.63 – 1.03). Stage 1 AKI was associated with 32% higher odds of HFpEF (OR 0.68, 95% CI 0.50 – 0.92) and stage 2–3 AKI was associated with 16% higher odds of HFrEF (OR 1.16, 95% CI 0.78 – 1.72).

#### Including imputed smoking status

In this analysis that used imputed data for smoking status, AKI (all stages) was associated with 21% higher odds of HFpEF (OR 0.79, 95% CI 0.62 – 1.00). Stage 1 AKI was associated with 34% higher odds of HFpEF (OR 0.66, 95% CI 0.50 – 0.89) and stage 2–3 AKI was associated with 10% higher odds of HFrEF (OR 1.10, 95% CI 0.75 – 1.62).

#### Excluding patients with HF with mildly reduced EF (HFmrEF)

In this analysis that was limited to patients with EF < 40% or ≥ 50%, AKI (all stages) was associated with 22% higher odds of HFpEF (OR 0.78, 95% CI 0.60 – 1.03). Stage 1 AKI was associated with 39% higher odds of HFpEF (OR 0.61, 95% CI 0.43 – 0.87) and stage 2–3 AKI was associated with 25% higher odds of HFrEF (OR 1.25, 95% CI 0.81 – 1.92).

#### Analysis stratified by sex

To evaluate for differences in outcomes by sex, we first replicated the primary analysis in each sex. Among men, AKI (all stages) was associated with 27% higher odds of HFpEF (OR 0.73, 95% CI 0.53 – 0.99). Stage 1 AKI was associated with 45% higher odds of HFpEF (OR 0.55, 95% CI 0.38 – 0.81), while stage 2–3 AKI was associated with 30% higher odds of HFrEF (OR 1.30, 95% CI 0.78 – 2.18). Among women, AKI (all stages) was associated with 8% higher odds of HFpEF (OR 0.92, 95% CI 0.64 – 1.32). Stage 1 and stages 2–3 AKI were both associated with 8% higher odds of HFpEF (stage 1 OR 0.92, 95% CI 0.59 – 1.43; stage 2–3 OR 0.92, 95% CI 0.50 – 1.67.) We included a sex-by-AKI interaction term in the model to evaluate for effect modification by sex, which was not statistically significant.

#### Analysis of weighted cohort

As an additional sensitivity analysis, we used inverse probability of treatment weights (IPTW) to synthesize a cohort that could match the HFrEF patients to the HFpEF on all observed covariates except for AKI, as would be done in a case–control study with a sufficient number of controls to allow such matching. Characteristics of the weighted cohort are detailed in Table 3. In this analysis, AKI (all stages) was associated with 20% higher odds of HFpEF (OR 0.80, 95% CI 0.60 – 1.05). Stage 1 AKI was associated with 33% higher odds of HFpEF (OR 0.67, 95% CI 0.48 – 0.93) and stage 2–3 AKI was associated with 14% higher odds of HFrEF (OR 1.14, 95% CI 0.72 – 1.82).

**Table 3:** Baseline characteristics of weighted cohort

Variable	HFpEF	HFrEF
Count (W)	1839	1842
Age at heart failure: Mean (SD)	64.67 (14.72)	64.73 (14.60)
Female Sex at Birth: % (W)	39.7% (730)	39.5% (728)
Race: % (W)		
Black	17.1% (314)	17.0% (314)
Other^a^	2.3% (42)	2.3% (43)
White	80.7% (1484)	80.6% (1486)
Albumin: Mean (SD)	4.00 (0.45)	4.0 (0.46)
Systolic Blood Pressure: Mean (SD)	133.40 (21.23)	133.27 (21.17)
Diastolic Blood Pressure: Mean (SD)	76.05 (13.46)	76.06 (13.68)
Body Mass Index: Mean (SD)	29.37 (7.08)	29.39 (7.11)
Estimated GFR: Mean (SD)	83.78 (28.91)	83.44 (28.78)
Atrial Fibrillation: % (W)	13.7% (252)	13.6% (249)
Anemia: % (W)	7.3% (134)	7.2% (132)
COPD: % (W)	15.6% (286)	15.4% (283)
Coronary Artery Disease: % (W)	33.9% (623)	34.0% (627)
Diabetes: % (W)	30.2% (555)	30.3% (558)
Hypertension: % (W)	60.4% (1112)	60.4% (1113)
Liver Disease: % (W)	4.4% (81)	4.4% (81)
Myocardial Infarction: % (W)	9.8% (181)	9.8% (181)
Peripheral Artery Disease: % (W)	8.1% (149)	8.1% (149)
Rheumatic Disease: % (W)	6.0% (111)	6.0% (111)
Alcohol Abuse: % (W)	3.4% (63)	3.4% (63)
Cancer: % (W)	23.4% (430)	23.3% (429)
HIV: % (W)	2.1% (38)	2.1% (39)
Dyslipidemia: % (W)	70.0% (1281)	70.0% (1285)
CT Scan with Contrast: % (W)	1.7% (32)	1.7% (31)
Ace Inhibitors: % (W)	19.0% (348)	19.1% (352)
Alpha Blockers: % (W)	1.2% (22)	1.2% (23)
Beta Blockers: % (W)	20.0% (359)	19.7% (364)
Loop Diuretics: % (W)	7.5% (138)	7.6% (140)
Mineralocorticoid Antagonists: % (W)	1.6% (30)	1.7% (31)
Nitrates: % (W)	7.2% (133)	7.2% (132)
Non-Loop Diuretics: % (W)	14.3% (263)	14.3% (264)
Statins: % (W)	24.5% (451)	24.7% (455)
NSAIDS: % (W)	17.1% (314)	17.0% (213)
Calcium Channel Blockers: % (W)	15.6% (287)	15.7% (289)
Angiotensin II Receptor Antagonists: % (W)	9.9% (182)	9.9% (183)
Insulin: % (W)	6.1% (112)	6.1% (112)
Metformin: % (W)	8.9% (163)	8.9% (164)
Other Diabetes Medications: % (W)	7.8% (144)	7.9% (146)

W is the sum of the patient weights, which is the effective number of patients in the weighted cohort created by matching weights

All *p*-values are > 0.80

^a^Other Race includes Asian, Alaskan/Indian, Native Hawaiian/Pacific Islander, unknown, or declined to answer

### Post hoc analysis

Given the unexpected opposite directionality of the point estimates for stage 1 AKI compared to stages 2–3 AKI, we conducted a post hoc analysis to test for a unique additional effect of severe AKI in contrast to the overall AKI effect. In this analysis, the overall AKI effect (all AKI stages) was associated with 32% higher odds of HFpEF (OR 0.68, 95% CI 0.51 – 0.91), while the additional severe AKI effect (stage 2–3 AKI) was associated with 64% higher odds of HFrEF which was statistically significant (OR 1.64, 95% CI 1.03 – 2.63).

We also examined the number of MI events in each group to determine if a larger proportion of MIs during or after stage 2–3 AKI could explain the risk of HFrEF. Of the 460 patients with AKI, 39 had an MI (defined by presence of both a diagnosis code for myocardial infarction and elevated troponin level) during or within 5 days of the AKI hospitalization; of these, 31 developed HFpEF and 8 developed HFrEF. Similarly, 25% of those with stage 1 AKI and 20% of those with stage 2–3 AKI had an MI in the time between the AKI event and the date of HF.

## Discussion

AKI has been shown to be strongly associated with HF in prior studies [10, 11]. The nature of this association and whether AKI is preferentially associated with the type of HF observed has not been previously studied. To our knowledge, this is the first study to examine the nature of HF experienced by AKI survivors. In this study, we found a statistically significant association between stage 1 AKI and HFpEF. Stages 2–3 AKI did not demonstrate a significant association with HF subtype, although point estimates trended towards HFrEF (ORs > 1). The association we found between stage 1 AKI and HFpEF is consistent with other known risk factors for HFpEF such as older age, female sex, obesity, and atrial fibrillation [5, 14–16].

There are multiple potential explanations for our findings. As multimorbidity is a risk factor for HFpEF [17], the association between stage 1 AKI and HFpEF could reflect a comorbidity burden that both increases an individual’s risk of HFpEF and causes a decreased renal reserve that renders them more likely to have small fluctuations in serum creatinine during illness or hospitalization. Alternatively, rather than simply functioning as an indicator of multimorbidity and consequent risk of HFpEF, it is possible that mild AKI may mediate a direct effect on the heart. It is well established in preclinical studies that AKI adversely affects the heart and leads to echocardiographic evidence of cardiac dysfunction [18–20] including diastolic dysfunction [8, 9]. In the ischemia reperfusion injury (IRI) model, a single episode of transient AKI leads to diastolic dysfunction by three days [8] which persists up to one year [8, 9]. Preclinical data also provides insights on potential mechanisms of AKI-induced cardiac dysfunction, showing that AKI is associated with cardiomyocyte apoptosis, cardiac inflammation, mitochondrial dysfunction, and reduced ATP levels [21]. As both diastolic and systolic function are energy requiring processes, mitochondrial dysfunction leading to inadequate ATP levels is a plausible explanation of diastolic dysfunction in murine models (especially given the absence of traditional causes of diastolic dysfunction such as hypertension and severe acidosis) [8, 9]. A specific circulating mediator of AKI-induced cardiac dysfunction has not yet been identified, although proinflammatory cytokines and uremic toxins may play a role [20, 22]. Since AKI is known to induce systemic inflammation [23], which is a major driver of HFpEF pathogenesis [2], AKI-induced inflammation is a plausible potential mediator of the association with HFpEF.

Our findings related to more severe AKI were unexpected. While our small sample size may have precluded our ability to detect a statistically significant association between severe AKI and HFrEF, the opposite directionality of the point estimate for stage 2–3 AKI (compared to stage 1) could suggest a different or unique mechanism with more severe AKI. The post hoc analysis that modeled an overall AKI association (as could be caused by a multimorbidity effect) and an additional severe AKI association (as could be caused by a separate mechanism unique to stage 2–3 AKI) found a significant association unique to stage 2–3 AKI for HFrEF. Such an association could be explained by higher illness severity that results in a decreased ejection fraction (i.e., severe AKI occurring after a massive MI with cardiogenic shock). However, while administrative codes do not capture disease severity, the MI events that occurred during the AKI hospitalization overwhelmingly occurred in patients who developed HFpEF, so it seems unlikely that co-occurrence of MI and stage 2–3 AKI entirely explains the association. It is also possible that complications of or therapy for severe AKI may increase the risk of HFrEF, as both uremia and acidosis have been shown to affect cardiac function in preclinical studies [22, 24], and hemodialysis can cause myocardial stunning [25]. Another potential explanation is AKI worsening preexisting cardiac injury. A recent study found that AKI dramatically worsened pre-existing cardiac injury in rats, resulting in global cardiac dysfunction including reduced left ventricular systolic function [26]. Future studies of HF risk after severe AKI events with larger sample sizes are needed to investigate this potential association further.

Strengths of this study include a creatinine-based definition of AKI, clinical adjudication of AKI cases classified as stage 3 based on dialysis (to ensure CKD progression was not misclassified as AKI), an outcome defined using echocardiogram data in addition to administrative codes, and a dataset enriched for patients obtaining longitudinal care within the health system. Limitations include a predominantly White population, which may limit generalizability to more diverse populations, and inclusion of only inpatient AKI, which may underestimate number of AKI events. While detailed phenotyping of AKI subtype (i.e., acute tubular injury, obstructive AKI, etc.) was not feasible, we modeled stage 1 versus stage 2–3 AKI, as stage 2–3 AKI is more likely to represent parenchymal damage. Future work that enables more granular AKI phenotyping in EHR data will facilitate investigations of the associations between different forms of AKI with HF. It is also possible that some patients may have developed HF that was diagnosed outside of the Vanderbilt system, however by requiring 3 outpatients visits within 5 years in our inclusion criteria, we enriched our dataset for patients receiving longitudinal care within our health system and thus limited the likelihood that patients would have been diagnosed with HF outside of the Vanderbilt health system. Additionally, as our objective in this study was to test for a preferential association of AKI with HFpEF or HFrEF, we did not seek to recapitulate the risk of HF after AKI, which has been previously demonstrated [10, 11]. We also defined the HF outcome with an EF cutoff of ± 45%, rather than the current American College of Cardiology definitions (HFrEF < 40%, HFmrEF 40–49%, HFpEF ≥ 50%) [27], as the pathophysiology underpinning HFmrEF is less well-defined and use of a binary outcome allowed us to leverage the case–control study design to test for a preferential association between AKI and HF subtype. However, results of the sensitivity analysis excluding patients with HFmrEF were consistent with the primary analysis, which is reassuring that our choice of a 45% EF cutoff did not materially affect our findings. Finally, we cannot infer causation due to the observational nature of this study.

In summary, we found that while AKI overall did not have a preferential association with HFrEF or HFpEF, stage 1 AKI had a statistically significant association with HFpEF. Our findings underscore the importance of close follow-up and attention to volume status and cardiac function after a hospitalization complicated by even mild AKI.

## Data Availability

The data that support the findings of this study are not publicly available due to the inclusion of confidential electronic health record data. Data is available from the Vanderbilt University Medical Center Synthetic Derivative for researchers who meet the criteria for access, which requires a data use agreement. Contact evan.brittain@vumc.org to request the data from this study.
